# Serum Bile Acid Profiles in Latent Autoimmune Diabetes in Adults and Type 2 Diabetes Patients

**DOI:** 10.1155/2022/2391188

**Published:** 2022-02-22

**Authors:** Yu Zhou, Deli Ye, Xiaofen Yuan, Yonglie Zhou, Jun Xia

**Affiliations:** ^1^Department of Clinical Laboratory, Zhejiang Provincial People's Hospital, People's Hospital of Hangzhou Medical College, Zhejiang, 310014 Hangzhou, China; ^2^Hangzhou Calibra Diagnostics Co., Ltd, Gene Town, Zijin Park, 859 Shixiang West Road, Xihu District, Hangzhou, Zhejiang, China

## Abstract

**Background:**

Impaired bile acid (BA) metabolism has been associated with the progression of type 2 diabetes (T2D). However, the contribution of BAs to the pathogenesis of latent autoimmune diabetes in adults (LADA) remains unclear. This study was aimed at investigating the association of serum BAs with different diabetes types and analyzing its correlation with main clinical and laboratory parameters.

**Methods:**

Patients with LADA, patients with T2D, and healthy controls (HCs) were enrolled. Serum BA profiles and inflammatory cytokines were measured. The correlation of BA species with different indicators was assessed by Spearman's correlation method.

**Results:**

Patients with diabetes (LADA and T2D) had significantly higher serum BAs, especially conjugated BAs, compared with those in HCs. Nevertheless, serum BA profiles had no special role in the progression of LADA, because no significant differences in BAs were observed between LADA and T2D patients. Interestingly, HbA1c levels and HOMA-*β* were found to be correlated with a series of BA species. Proinflammatory cytokines (IL-1*β*, IL-6, and TNF-*α*) and anti-inflammatory cytokine (IL-10) were all positively associated with several BA species, especially the conjugated secondary BAs.

**Conclusion:**

Serum BAs regulate glucose homeostasis, but have no special value in the pathogenesis of LADA patients. Our study adds further information about the potential value of serum BAs in different types of diabetes.

## 1. Introduction

Diabetes is a major threat to global public health. Since it is a complex disease, it cannot be simply subdivided into type 1 diabetes (T1D) and type 2 diabetes (T2D) [1]. The latent autoimmune diabetes in adults (LADA), also known as type 1.5 diabetes, is a common but understudied subtype of diabetes. According to reports, LADA accounts for 2–12% of all types of diabetes, with higher incidence in Northern Europe and China (7–14%) [2, 3]. Given that LADA is a hybrid of T1D and T2D clinically and metabolically, it has considerable heterogeneity [4]. As the most common form of diabetes, T2D accounts for 90–95% of all diabetic cases worldwide [5]. However, due to the overlap of clinical characteristics, approximately 5.9% of newly diagnosed T2D cases in Chinese population are actually misdiagnosed LADA [6]. Therefore, for more accurate diagnosis and treatment, studies regarding the differences of underlying pathophysiology between LADA and T2D are urgently needed.

Growing evidences have revealed that impaired bile acid (BA) metabolism likely contributes to the pathophysiology of metabolic diseases, such as diabetes [7]. BAs are amphipathic molecules derived from cholesterol, which can promote the digestion and absorption of lipids, regulate cholesterol metabolism, and promote bile secretion [8]. According to reports, BAs can also regulate a variety of cellular functions, such as inhibiting NLRP3 inflammasome [9] and regulating immune cells [10, 11].

Inflammatory is the major contributor in the development and progression of diabetes. Some serum immune mediators such as low-grade proinflammatory markers and cellular immunology have been found to increase in different types of diabetes [12, 13]. BAs are signaling molecules that coordinately regulate metabolism and inflammation via nuclear Farnesoid X receptor (FXR) and Takeda G protein-coupled receptor 5 (TGR5), both of which have a broader range of metabolic effects on insulin sensitivity, inflammation, and glucose control [14]. Although it has been reported that serum BAs are significantly associated with the high risk of T2D [15, 16], the correlation between serum BA spectrum and different types of diabetes has not been reported. In this study, we analyzed the level and composition of serum BAs in patients with different types of diabetes, in order to explore the potential value of BAs in the underlying pathogenesis of LADA and T2D patients. Our study can further clarify the role of BA spectrum in the different course of diabetes.

## 2. Materials and Methods

### 2.1. Participants

In this study, patients diagnosed with LADA (*n* = 35) were included from July 2020 to September 2021. Gender- and age-matched patients with T2D (*n* = 69) and healthy controls (HCs, *n* = 50) were randomly selected in the same period. The study protocol was approved by the ethics committee of Zhejiang Provincial People's Hospital, and written informed consent was obtained. Diabetes diagnosis was conformed to the World Health Organization (WHO) 1999 criteria. The definitions of LADA were as follows: (1) no ketosis or ketoacidosis, (2) insulin independence for the first 6 months after diabetes diagnosis, (3) at least one positive islet autoantibody (glutamic acid decarboxylase 65 antibody (GADA) or insulinoma-associated protein-2 antibody (IA-2A)), and (4) age ≥ 30 years at onset of diabetes. The criteria for T2D were negative for islet autoantibodies and no immediate insulin treatment requirement. Exclusion criteria were as follows: (1) secondary diabetes mellitus, (2) pregnant women, (3) malignant disease, (4) acute infection, or (5) receiving immunosuppressive. HCs were drawn from healthy individuals who underwent physical examination in our hospital.

### 2.2. Clinical and Biochemical Parameters

Clinical characteristics include gender, age, body mass index (BMI), systolic blood pressure (SBP), diastolic blood pressure (DBP), diabetes duration, and family history of diabetes; laboratory parameters including diabetes-unrelated autoantibodies (thyroid peroxidase (TPO) antibody and thyroglobulin (Tg) antibody), glycemic parameters (glycosylated hemoglobin (HbA1c), fasting plasma glucose (FPG), and fasting C-peptide (FCP)), liver function-related indicators (total bilirubin (TBIL) and *γ*-glutamyl transpeptidase (GGT)), kidney function-related indicators (creatinine (CREA) and UREA), and lipid profiles (triglycerides (TG), total cholesterol (TC), low-density lipoprotein cholesterol (LDL-C), and high-density lipoprotein cholesterol (HDL-C)) were recorded. The above routine lab indicators were measured using the autoanalyzer. The homeostasis models assessment of insulin resistance (HOMA-IR) index and *β* cell function (HOMA-*β*) were also calculated [17].

### 2.3. BA Measurement and Classification

The peripheral blood samples of all participants were collected under fasting conditions. After centrifugation, the sera were stored at −80°C and thawed until testing. Serum BAs were analyzed using the liquid chromatography coupled to tandem mass spectrometry (LC-MS/MS, AB SCIEX Triple Quad™ 4500MD), which provided precise quantification and extensive coverage of essential BA species. BA spectrums were extracted according to the manufacturer's instruction (DISIGNS Diagnostics). The corresponding quality control and standard products were employed at the same time. For BA concentrations below standard range and out of invertible range, 0.00 *μ*mol/L was assigned. Subgrouping of the measured BAs, ratios reflective of enzymatic activities in the liver (TCA/CA, GCA/CA, TCDCA/CDCA, and GCDCA/CDCA), 12a-OH BA species (CA, DCA, and their conjugates), and 12a-OH/non12a-OH ratios were referred to the study of Lu et al. [15]. Meanwhile, the hydrophobicity index (HI) of the measured BAs was also calculated using the published data [18].

### 2.4. Cytokine Assays

The collected serum samples were simultaneously analyzed for inflammatory cytokines. The concentration and median fluorescence intensity (MFI) of IL-1*β*, IL-6, IL-10, and TNF-*α* were measured using the BD Cytometric Bead Array (CBA) Human Soluble Protein Flex Set System. Data analyses were performed through FCAP Array v3.0.

### 2.5. Statistical Analysis

In order to describe the characteristics of participants, the mean ± SD or median (interquartile range, IQR) were used for continuous variables. Frequencies were used for categorical variables. Differences of clinical and laboratory data between groups were analyzed using Student's *t*-test, ANOVA, or Wilcoxon rank-sum test where appropriate. The Fisher's exact test or chi-square (*χ*^2^) test was used for categorical data with two or more classes. Data on these analyses were not adjusted for multiple comparisons and were therefore descriptive. GraphPad Prism 8.4.2 and SPSS 22.0 were applied for statistical analyses. *P* < 0.05 was considered statistically significant. Spearman's correlation was used to correlate the serum BA levels of all participants with different parameters.

## 3. Results

### 3.1. Baseline Characteristics of Participants

The clinical and laboratory characteristics of participants (35 LADA patients, 69 T2D patients, and 50 HCs) are shown in Table 1. All three groups were similar in gender and age characteristics, but patients with diabetes (LADA and T2D) tended to have higher levels of SBP, DBP, and BMI (all *P* < 0.05). As for laboratory indicators, there were significant differences among the three groups of thyroid-associated autoantibodies (TPO and Tg), liver function-related indicator (GGT), lipid profiles (TG, LDL-C, and HDL-C), glycemic parameters (HbA1c, FPG, and FCP), and HOMA-*β* (all *P* < 0.05). However, the clinical and laboratory indicators between LADA and T2D patients were basically the same, except for family history of diabetes (*P* = 0.033), HDL-C value (*P* = 0.018) and the positive rate of TPO (*P* = 0.029), and Tg (*P* = 0.003).

As more and more evidence suggest that LADA patients comprise a highly heterogenous group of patients [19, 20], subgroup analyses of LADA patients based on the number of positive diabetes-related autoantibodies, GADA titers, and C-peptide levels were also performed (Table 2). Among LADA patients, more than half were GADA positive, but most of them had low GADA titers (<200 IU/mL). Meanwhile, 65.71% of LADA patients had a C − peptide level > 0.7 nmol/L, and only 5.71% showed low level of C-peptide secretion (<0.3 nmol/L).

### 3.2. Distribution of BAs in the Study Population

**Figure 1:**
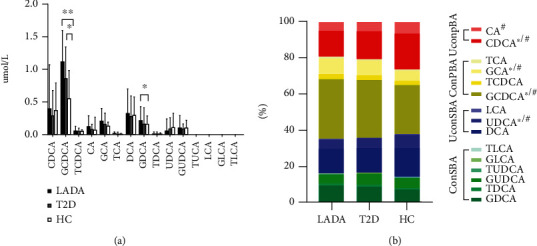
Distribution of serum BAs in LADA patients, T2D patients, and healthy controls. (a) Serum levels of BAs in the study population. Due to lack of normality, results were expressed as median and interquartile range. ^∗^*P* < 0.05; ^∗∗^*P* < 0.01. (b) The composition of BAs in the study population. ^∗^*P*_LADA vs.HC_ < 0.05; ^#^*P*_T2D vs.HC_ < 0.05.

Mass spectrometry results showed that GCDCA and CDCA were the largest contributors to serum BA levels, while the TCA, TDCA, TUDCA, LCA, GLCA, and TLCA were relatively low (Figure 1(a)). Interestingly, there was no significant difference in the absolute concentration (Figure 1(a)) and composition (Figure 1(b)) of BAs between LADA and T2D patients. Meanwhile, the level of GCDCA in subjects with diabetes was significantly higher compared with HCs (*P*_LADA vs.HC_ = 0.001 and *P*_T2D vs.HC_ = 0.025); the GDCA level of LADA patients was higher than that of the HC group (*P* = 0.025). The composition of individual BAs was statistically different in diabetics compared with control subjects. Among them, LADA and T2D patients had a higher percentage of GCA (*P*_LADA vs.HC_ = 0.016 and *P*_T2D vs.HC_ = 0.037) and GCDCA (*P*_LADA vs.HC_ = 0.023 and *P*_T2D vs.HC_ = 0.044), but a lower percentage of CDCA (*P*_LADA vs.HC_ = 0.028 and *P*_T2D vs.HC_ = 0.013) and UDCA (*P*_LADA vs.HC_ = 0.012 and *P*_T2D vs.HC_ = 0.027). Furthermore, the percentage of CA in T2D patients was significantly lower compared to HCs (*P* = 0.033).

To further assess the characteristics of BA profiles specific to different types of diabetes, BA subgroup concentrations were determined as well (Table 3). Although there was no statistical difference between LADA and T2D patients in the BA subgroup analyses, the concentration of total BAs (*P* = 0.018) and 12a-OH BA species (*P* = 0.012) were significantly increased in LADA patients compared with those in HCs. Serum total glycine-conjugated BAs were significantly higher in LADA (*P* < 0.001) and T2D (*P* = 0.017) subjects compared with the control group; in contrast, there was no significant difference in the absolute concentration of taurine-conjugated BAs among the three groups. The BA subgroup analyses also revealed that the ratios of Unconj./Conj. BAs (*P*_LADA vs.HC_ = 0.004 and *P*_T2D vs.HC_ = 0.002), GCA/CA (*P*_LADA vs.HC_ = 0.024 and *P*_T2D vs.HC_ = 0.003), and GCDCA/CDCA (*P*_LADA vs.HC_ = 0.010 and *P*_T2D vs.HC_ = 0.006) were significantly different in diabetics compared to HCs.

### 3.3. Circulating Cytokines in Different Diabetes Types

**Figure 2:**
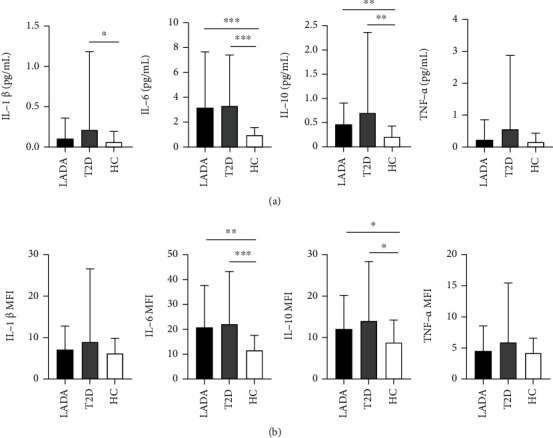
Circulating cytokines in LADA patients, T2D patients, and healthy controls. Results were expressed as the mean ± SD. (a) Cytokine concentrations in the different cohorts. (b) MFI of different cytokines in the participants. ^∗^*P* < 0.05, ^∗∗^*P* < 0.01, and ^∗∗∗^*P* < 0.001.

As shown in Figure 2, the concentration (Figure 2(a)) and MFI (Figure 2(b)) of IL-6 and IL-10 were significantly elevated in diabetics than those in HC individuals (all *P* < 0.05). Additionally, the level of IL-1*β* in T2D patients was higher than that in the HC group (*P* = 0.025). Although T2D patients tended to have higher levels of TNF-*α*, they did not reach a statistical difference.

### 3.4. Correlation of BAs with the Main Clinical and Laboratory Parameters

**Figure 3:**
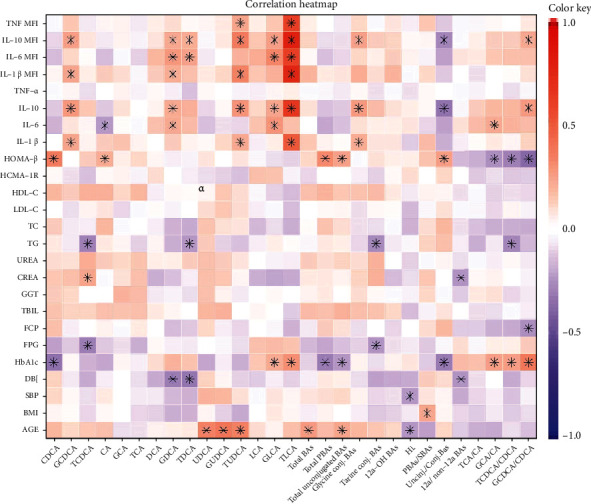
Correlation study of the association of BAs with the main clinical and laboratory Parameters. The color keys represent the regression coefficients of the independent variables. PBA: primary BA; SBA: secondary BA; Unconj.: unconjugated; Conj.: conjugated. ^∗^*P* < 0.05.

When considering all participants together, Spearman correlation analyses (Figure 3) revealed that the concentration of total BAs, unconjugated BAs, UDCA, and its conjugated type were increased with age, while the HI value was inversely correlated. The ratio of PBAs/SBAs was significantly positively associated with BMI. Meanwhile, SBP showed negative correlation with HI; DBP was negatively associated with 12*α*/non-12*α* BAs, GDCA, and TDCA. Notably, the HbA1c level and HOMA-*β* were related to a series of BA indexes, but the trend was opposite. Total PBAs, unconjugated BAs, and Unconj./Conj. BAs were inversely correlated with HbA1c but positively associated with HOMA-*β*; the ratio reflective of enzymatic activities in the liver (GCA/CA, TCDCA/CDCA, and GCDCA/CDCA) increased with HbA1c whereas decreased with HOMA-*β*. Additionally, all measured serum BAs were found not related to HOMA-IR. Interestingly, proinflammatory cytokines (IL-1*β*, IL-6, and TNF-*α*) and anti-inflammatory cytokine (IL-10) had a significant positive association with several BA species, especially conjugated SBAs. TLCA level was significantly correlated with the MFI of all circulating cytokines detected. Furthermore, the ratio of Unconj./Conj. BAs was negatively correlated with IL-10.

## 4. Discussion

A better understanding of the factors that contribute to different diabetes courses is crucial for prevention and intervention strategies. Our study was to preliminarily explore the value of serum BAs in the pathogenesis of different diabetes types. The comparison of BA profiles in the serum of LADA cases, T2D cases, and healthy subjects was performed. Notably, changes in serum BAs were observed in patients with diabetes, but no significant differences were found between LADA and T2D patients. It was observed that HbA1c and HOMA-*β* levels were correlated with a range of BA species, but the trends were opposite. Moreover, both proinflammatory and anti-inflammatory cytokines were positively associated with several BA species, especially conjugated SBAs.

As expected, clinical and laboratory features related to diabetes were observed in patients with LADA and T2D. However, there was no statistical difference in the insulin resistance (HOMA-IR) index among the three groups. On average, LADA patients have fewer diabetic dyslipidemia but higher frequency of autoimmune diseases [21]. In line with the above statement, we found that HDL-C value and the positive rate of thyroid-associated autoantibodies (TPO and Tg) were higher in LADA patients than T2D patients. However, other general characteristics of LADA patients were not observed in this study, including lower BMI, fasting C-peptide, and blood pressure disorders [6, 22]. Among autoantibodies against *β* cells, GADA is the most common marker in LADA patients. Both Swedish and Norwegian data have demonstrated that differences in GADA titers can affect the phenotype of LADA patients [23]. Meanwhile, according to the latest expert consensus on LADA management, C-peptide level was introduced to drive the therapeutic decisions for LADA patients [21]. When the C-peptide level is >0.7 nmol/L, it is recommended to use a slightly modified ADA/EASD algorithm for T2D. In our study, 82.86% of the enrolled LADA individuals had low GADA titers, and only 5.71% showed low levels of C-peptide secretion, which further implicated that the clinical characteristics of LADA and T2D patients were highly consistent in this study.

Recently, increasing studies have shown that BAs may regulate glucose tolerance, insulin sensitivity, and energy metabolism [24, 25]. Our study helps to systematically evaluate the changes of serum BA profile in different courses of diabetes. Intriguingly, there was no significant difference in the absolute concentration and composition of BAs between LADA and T2D patients, indicating to some extent that the autoimmune response in LADA patients may not affect the composition of serum BA spectrum. Nonetheless, significant differences were observed between subjects with diabetes and healthy individuals. In this study, the total BA concentration in patients with diabetes was higher, especially in LADA patients, which supports the view that total BA concentration tends to increase in onsetting diabetes [24]. Furthermore, the concentration of conjugated BAs (GCDCA and GDCA), which associated with increased risk of diabetes [15, 26], was significantly elevated in diabetics. The composition of BAs and the ratio between conjugated to unconjugated primary BAs (Unconj./Conj. BAs, GCA/CA, and GCDCA/CDCA) indicated that LADA and T2D patients had an excessive transition from unconjugated primary BAs to the conjugated, which is considered to play an essential role in the pathogenesis of T2D development [15]. It has been demonstrated that the sum of 12*α*-OH BA increases with the increasing insulin resistance [27]. Similarly, we found that subjects with diabetes tended to have higher concentrations of 12*α*-OH BA. The relative hydrophobicity of BA is an important determinant of bioactivity [28]; however, there was no statistical difference in the HI among the three groups.

Since chronic inflammation modulates liver BA metabolism, circulating cytokines were also analyzed. Consistent with our previous results [29], the level of IL-6 and IL-10 in patients with diabetes was significantly elevated than those in HC individuals. Nevertheless, there was no significant difference in the cytokine changes between LADA and T2D patients in the present study. Given that the IL-1*β*, IL-6, and TNF-*α* are proinflammatory cytokines related to insulin resistance [30], the high similarity of HOMA-IR index between LADA and T2D in this study can explain the above ambiguity to a certain extent.

In line with the results of Zhu et al. [16], the total BA concentration was positively correlated with age. It has been reported that insulin resistance indicated by HOMA-IR may partially mediate the association between BAs and T2D [31]. However, we observed that several BA species were correlated with *β* cell function index HOMA-*β*, rather than HOMA-IR. This discrepancy may be partly due to epidemiological features (in this study, there was no statistical difference in HOMA-IR among the three cohorts) and study design (not only T2D and HC individuals, but also LADA patients were studied in the current study). HbA1c, as the universally accepted standard for the diagnosis and monitoring of diabetes [32], was found to be negatively correlated with total primary BAs, unconjugated BAs, and Unconj./Conj. BAs. This phenomenon indicated that the imbalance of unconjugated vs. conjugated BAs or of secondary vs. primary BAs may play an important role in the process of diabetes.

The dual function of BAs in inflammation has been reported previously. Some studies have shown that activation of the BA responsive receptor TGR5 mediates anti-inflammatory effects [9, 33]. Other reports demonstrate that BAs can induce the excretion of proinflammatory cytokines such as IL-1*β* and TNF-*α* in hepatic macrophages [34, 35]. Our study revealed that several BA species, especially conjugated SBAs, were positively associated with circulating cytokines, including the proinflammatory cytokines (IL-1*β*, IL-6, and TNF-*α*) and anti-inflammatory cytokine (IL-10). The dual role of BAs in regulating cytokine expression suggests that they can modulate inflammatory response through different mechanisms, and the exact mechanism involved remains to be further studied.

This study also had some limitations. First of all, LADA patients can be further grouped according to certain indicators, but we did not analyze the distribution of BA profiles in LADA subgroups due to the limited sample size. Secondly, there is no uniform agreement on the definition of LADA, resulting in different application scopes of diagnostic criteria. Finally, some BA species that were significantly related to circulating cytokines, such as TLCA and TUDCA, had very low concentrations in the study population, which may affect the validity of results. Therefore, further researches are needed to fill these gaps.

## 5. Conclusions

Our results indicated that serum BAs regulate glucose homeostasis, but have no special role in the progression of LADA. Meanwhile, some laboratory parameters, especially circulating cytokines, were found correlated to a series of BA species. In fact, to our best knowledge, this is the first study to explore the association between serum BAs and different types of diabetes.

## Figures and Tables

**Table 1:** Comparison of clinical information and laboratory indicators in LADA patients, T2D patients, and healthy controls.

Characteristics	LADA (*n* = 35)	T2D (*n* = 69)	HC (*n* = 50)	*P* value			
				All	LADA vs. T2D	LADA vs. HC	T2D vs. HC
*Demographics*							
Male (*n*, %)	18 (51.43%)	42 (60.87%)	29 (58.00%)	0.654	0.357	0.549	0.753
Age (years) mean ± SD	52.37 ± 15.07	52.99 ± 13.81	51.68 ± 10.29	0.840	0.836	0.815	0.574
BMI (kg/m^2^) M (IQR)	24.51 (21.65, 27.71)	24.59 (22.66, 27.33)	22.71 (21.57, 25.06)	0.012^∗^	0.577	0.071	0.026^∗^
SBP (mmHg) mean ± SD	140.54 ± 22.78	136.33 ± 14.90	124.02 ± 16.74	0.001^∗∗^	0.504	0.002^∗∗^	0.001^∗∗^
DBP (mmHg) mean ± SD	82.14 ± 11.11	80.72 ± 9.71	73.40 ± 13.17	0.003^∗∗^	0.636	0.005^∗∗^	0.003^∗∗^
Diabetes duration (years) M (IQR)	3.50 (1.00, 12.00)	4.00 (0.75, 10.00)	NA	NA	0.692	NA	NA
Family history of diabetes (*n*, %)	18 (51.43%)	50 (72.46%)	NA	NA	0.033^∗^	NA	NA
*Diabetes-unrelated autoantibodies*							
TPO (>9 IU/ml; *n*, %)	6 (17.14%)	2 (2.90%)	7 (14.00%)	0.022^a^^∗^	0.029^a^^∗^	0.692	0.056^a^
Tg (>4 IU/ml; *n*, %)	10 (28.57)	5 (7.25%)	7 (14.00%)	0.013^∗^	0.003^∗∗^	0.098	0.227
*Other laboratory indicators*							
HbA1c (%) M (IQR)	9.75 (6.98, 11.85)	8.60 (7.20, 10.00)	5.20 (5.08, 5.50)	<0.001^∗∗∗^	0.449	<0.001^∗∗∗^	<0.001^∗∗∗^
FPG (mmol/L) M (IQR)	6.63 (5.48, 9.06)	7.55 (6.24, 9.37)	4.81 (4.49, 5.16)	<0.001^∗∗∗^	0.626	<0.001^∗∗∗^	<0.001^∗∗∗^
FCP (nmol/L) M (IQR)	1.16 (0.59. 2.32)	1.45 (0.87, 2.13)	2.16 (1.66, 2.63)	<0.001^∗∗∗^	0.241	<0.001^∗∗∗^	<0.001^∗∗∗^
TBIL (mg/dL) M (IQR)	11.60 (9.70, 14.05)	13.00 (11.30, 16.80)	13.65 (11.23, 16.93)	0.108	0.086	0.037^∗^	0.722
GGT (U/L) M (IQR)	26.00 (16.50, 70.00)	26.00 (20.00, 42.00)	22.00 (14.00, 34.00)	0.039^∗^	0.734	0.051	0.042^∗^
CREA (mg/dL) M (IQR)	73.30 (63.70, 83.35)	76.80 (65.60, 86.30)	79.15 (62.68, 93.98)	0.659	0.523	0.356	0.726
UREA (mg/dL) M (IQR)	5.01 (4.21, 6.23)	5.16 (4.34, 6.64)	5.26 (4.53, 5.92)	0.668	0.502	0.966	0.370
TG (mmol/L) M (IQR)	1.20 (0.76, 2.02)	1.38 (1.04, 2.19)	1.13 (0.83, 1.52)	0.008^∗∗^	0.226	0.356	0.001^∗∗^
TC (mmol/L) mean ± SD	4.62 ± 1.14	4.81 ± 1.08	5.07 ± 0.80	0.076	0.799	0.055	0.042^∗^
LDL-C (mmol/L) mean ± SD	2.40 ± 0.84	2.78 ± 0.84	2.94 ± 0.69	0.012^∗^	0.105	0.002^∗∗^	0.116
HDL-C (mmol/L) M (IQR)	1.17 (0.97, 1.41)	0.99 (0.86, 1.14)	1.34 (1.15, 1.58)	<0.001^∗∗∗^	0.018^∗^	0.003^∗∗^	<0.001^∗∗∗^
HOMA-IR M (IQR)	1.58 (0.56, 7.05)	2.05 (0.84, 3.24)	1.58 (1.29, 1.94)	0.536	0.427	0.523	0.306
HOMA-*β* M (IQR)	23.57 (10.10, 47.10)	26.22 (9.09, 55.45)	116.95 (84.58, 146.52)	<0.001^∗∗∗^	0.767	<0.001^∗∗∗^	<0.001^∗∗∗^

Data were shown as *n* (%), mean ± SD, or medians (interquartile range, IQR). BMI: body mass index; SBP: systolic blood pressure; DBP: diastolic blood pressure; TPO: thyroid peroxidase antibody; Tg: thyroglobulin antibody; HbA1c: glycosylated hemoglobin; FPG: fasting plasma glucose; FCP: fasting C-peptide; GGT: *γ*-glutamyl transpeptidase; CREA: creatinine; TG: triglycerides; TC: total cholesterol; LDL-C: low-density lipoprotein cholesterol; HDL-C: high-density lipoprotein cholesterol; NA: no analysis. ^a^Fisher exact test. ^∗^*P* < 0.05, ^∗∗^*P* < 0.01, and ^∗∗∗^*P* < 0.001.

**Table 2:** Subgroup analysis of 35 LADA patients.

Indexes	Number (%)
*Diabetes-related autoantibodies^a^*	
GADA (>10 IU/ml)	23 (65.71%)
IA-2A (>10 IU/ml)	15 (42.86%)
ICA (>1 COI)	17 (48.57%)
IAA (>1 COI)	15 (42.86%)
*GADA titers*	
High GADA titers (>200 IU/mL)	6 (17.14%)
Low GADA titers (<200 IU/mL)	29 (82.86%)
*C-peptide levels*	
<0.3 nmol/L	2 (5.71%)
≥0.3 and ≤0.7 nmol/L	10 (28.57%)
>0.7 nmol/L	23 (65.71%)

Data were expressed as *n* (%). GADA: glutamic acid decarboxylase 65 antibody; IA-2A: insulinoma-associated protein-2 antibody; ICA: islet cell antibody; IAA: insulin autoantibody. ^a^77.14% of LADA patients were positive for two or more diabetes-related antibodies.

**Table 3:** Serum bile acid subgroup concentrations in LADA patients, T2D patients, and healthy controls.

Species	LADA (*n* = 35)	T2D (*n* = 69)	HC (*n* = 50)	*P* value			
				All	LADA vs. T2D	LADA vs. HC	T2D vs. HC
Total BAs	3.52 (2.01, 6.11)	2.87 (1.92, 4.19)	1.98 (1.45, 4.10)	0.043^∗^	0.138	0.018^∗^	0.132
Total PBAs	1.91 (1.04, 4.62)	1.72 (0.99, 2.79)	1.20 (0.78, 2.67)	0.051	0.235	0.862	0.247
Total unconjugated BAs	1.00 (0.62, 2.34)	0.97 (0.61, 1.66)	0.98 (0.57, 2.05)	0.627	0.380	0.728	0.513
Glycine Conj. BAs	1.79 (1.06, 3.39)	1.39 (0.93, 2.54)	1.08 (0.71, 1.44)	0.002^∗∗^	0.122	<0.001^∗∗∗^	0.017^∗^
Taurine Conj. BAs	0.10 (0.06, 0.17)	0.10 (0.04, 0.18)	0.10 (0.05, 0.13)	0.395	0.223	0.258	0.925
12a-OH BAs	1.05 (0.66, 2.30)	0.87 (0.48, 1.66)	0.78 (0.48, 1.41)	0.045^∗^	0.068	0.012^∗^	0.446
HI	0.42 (0.35, 0.50)	0.44 (0.36, 0.49)	0.42 (0.36, 0.49)	0.949	0.959	0.789	0.798
PBAs/SBAs	1.83 (1.14, 2.54)	1.74 (1.04, 3.70)	1.48 (1.15, 2.64)	0.697	0.866	0.459	0.477
Unconj./Conj. BAs	0.63 (0.30, 1.14)	0.56 (0.31, 1.34)	1.15 (0.64, 1.50)	0.003^∗∗^	0.882	0.004^∗∗^	0.002^∗∗^
12*α*/non-12*α* BAs	0.66 (0.32, 1.01)	0.69 (0.22, 1.25)	0.67 (0.27, 0.95)	0.819	0.687	0.532	0.777
TCA/CA	0.14 (0.08, 0.32)	0.19 (0.06, 0.61)	0.11 (0.04, 0.30)	0.230	0.672	0.268	0.087
GCA/CA	1.99 (1.03, 3.79)	2.79 (0.87, 5.69)	1.21 (0.48, 2.42)	0.008^∗∗^	0.744	0.024^∗^	0.003^∗∗^
TCDCA/CDCA	0.14 (0.09, 0.38)	0.21 (0.05, 0.78)	0.12 (0.07, 0.24)	0.262	0.715	0.206	0.119
GCDCA/CDCA	2.74 (1.49, 6.58)	3.34 (1.03, 9.09)	1.72 (0.91, 2.79)	0.008^∗∗^	0.715	0.010^∗^	0.006^∗∗^

Due to lack of normality, the results of BA subgroups were expressed as medians and quartiles. HI: hydrophobicity index; PBAs: primary BAs; SBAs: secondary BAs; Unconj.: unconjugated; Conj.: conjugated. Ratios reflective of enzymatic activities in the liver: TCA/CA, GCA/CA, TCDCA/CDCA, and GCDCA/CDCA. ^∗^*P* < 0.05, ^∗∗^*P* < 0.01, and ^∗∗∗^*P* < 0.001.

## Data Availability

All data used to support the findings of this article are available from the corresponding author upon request.
